# Microbiological and Physico-Chemical Characteristics of Black Tea Kombucha Fermented with a New Zealand Starter Culture

**DOI:** 10.3390/foods12122314

**Published:** 2023-06-08

**Authors:** Boying Wang, Kay Rutherfurd-Markwick, Naran Naren, Xue-Xian Zhang, Anthony N. Mutukumira

**Affiliations:** 1 School of Food and Advanced Technology, Massey University, Auckland 0745, New Zealand; b.wang6@massey.ac.nz; 2 School of Health Sciences, College of Health, Massey University, Auckland 0745, New Zealand; k.j.rutherfurd@massey.ac.nz; 3 School of Natural Sciences, Massey University, Auckland 0745, New Zealand; n.naren@massey.ac.nz (N.N.); x.x.zhang1@massey.ac.nz (X.-X.Z.)

**Keywords:** kombucha, acetic acid bacteria, yeast, *Komagataeibacter rhaeticus*, *Zygosaccharomyces lentus*, *Debaryomyces prosopidis*

## Abstract

Kombucha is a popular sparkling sugared tea, fermented by a symbiotic culture of acetic acid bacteria (AAB) and yeast. The demand for kombucha continues to increase worldwide, mainly due to its perceived health benefits and appealing sensory properties. This study isolated and characterised the dominant AAB and yeast from a starter culture and kombucha broth after 0, 1, 3, 5, 7, 9, 11, and 14 days of fermentation at ambient temperature (22 °C). Yeast and AAB were isolated from the Kombucha samples using glucose yeast extract mannitol ethanol acetic acid (GYMEA) and yeast extract glucose chloramphenicol (YGC) media, respectively. The phenotypic and taxonomic identification of AAB and yeast were determined by morphological and biochemical characterisation, followed by a sequence analysis of the ribosomal RNA gene (16S rRNA for AAB and ITS for yeast). The changes in the microbial composition were associated with variations in the physico-chemical characteristics of kombucha tea, such as pH, titratable acidity, and total soluble solids (TSS). During fermentation, the acidity increased and the TSS decreased. The yield, moisture content, and water activity of the cellulosic pellicles which had developed at the end of fermentation were attributed to the presence of AAB. The dominant AAB species in the cellulosic pellicles and kombucha broth were identified as *Komagataeibacter rhaeticus.* The yeast isolates belonged to *Debaryomyces prosopidis* and *Zygosaccharomyces lentus*.

## 1. Introduction

Kombucha is a popular functional sparkling fermented tea beverage that has been consumed for more than 5000 years [1]. The taste of kombucha varies from slightly fruity, sour and fizzy to vinegary, depending on the fermentation conditions. For example, a longer fermentation time of kombucha can result in a sour taste [2]. The regular consumption of kombucha is perceived to confer numerous health-promoting benefits including antioxidants, antimicrobial, anti-carcinogenic, detoxification, antitumor, and antihypertensive activities [2,3,4,5]. However, most of the perceived health benefits are based on in vitro models. Therefore, in vivo clinical trials are necessary to confirm the functional activities.

Kombucha is usually fermented from sucrose-sweetened black or green tea with an undefined symbiotic culture of bacteria and yeast (SCOBY) embedded in a cellulosic pellicle at an ambient temperature for 7–14 days [6,7,8]. Other tea varieties such as oolong tea, thyme, lemon balm, peppermint, rosemary, wheat grass, guava and oak leaves, fruit juices, milk, and laver have also been used to make kombucha [5,9,10,11]. During fermentation, sucrose is firstly hydrolysed by yeast to glucose and fructose. The glucose produced is then metabolised into gluconic and glucuronic acids by the AAB, whereas fructose is metabolised by yeast to produce ethanol and carbon dioxide. The resulting alcohol is further metabolised by the AAB into acetic acid. After fermentation, kombucha contains a complex chemical composition and favourable bioactive components including organic acids (acetic, gluconic, lactic, and glucuronic acids), polyphenols (epicatechin, epigallocatechin, epicatechin gallate, and epigallocatechin gallate), vitamins (B_1_, B_2_, B_6_, B_12_, and C), minerals (Cu, Fe, Mn, Ni, and Zn), ethanol, and amino acids [3,9,12]. The variations in the chemical and nutritional metabolites present in kombucha may be attributed to the initial concentration of starter culture added, to its diverse microbial composition, as well as to the amount of sugar (mostly sucrose), the fermentation conditions (temperature and time), and the type of tea used [2,13].

The microbial composition of kombucha is diverse and varies between fermentations, but is mainly composed of AAB and yeast [14]. Previous studies indicate that gluconic-acid-producing AAB are the dominant bacteria in the kombucha culture [15]. The most common AAB include *Komagataibacter* (*K.*) *kombuchae, K. saccharivorans, K. rhaeticus, K. intermedius, K. europaeus, K. hansenii*, and *K. xylinus*; *Gluconobacter* (*G.*) *oxydans, G. potus; Gluconacetobacter* (*G.*) *sacchari*, and *G.* sp A4; *Acetobacter* (*A.*) *musti*, *A. pasteurianus, A. aceti*, and *A. nitrogenifigens* sp. nov. [4,12,16,17]. Small amounts of lactic acid bacteria (LAB) have also been reported in kombucha [6], including *Lacticaseibacillus casei, Lactiplantibacillus plantarum, Lactobacillus rhamnosus, Lactobacillus mali, Pediococcus pentosaceus, P. acidiliactici, Liqourilactobacillus nagelii*, *Oenococcus oeni*, and *Bifidobacterium* [18,19,20,21,22,23]. There is scant information on the role of LAB in kombucha, thus, this is an area for future study. The most commonly reported yeast species in kombucha are *Brettanomyces* (*B.*) *bruxellensis, B. lambicus, B. intermedius; Candida* (*C.*) *albican, C. kefir, C. zemplinina; Dekkera* (*D.*) *bruxelensis*, and *D. anomala; Hanseniaspora* (*H.*) *valbyensis; Pichia* (*P.*) *fermentans, P. occidentalis*, and *P. kudriavzevii; Saccharomyces* (*S.*) *cerevisiae; Schizosaccharomyces* (*S.*) *pombe;* and *Zygosaccharomyces* (*Z.*) *bailii* and *Z. rouxii* [13,24,25,26,27,28]. The interactions between the bacteria and yeast produce kombucha with different chemical compositions [29].

The unique cellulose-forming characteristics of kombucha may be useful for a variety of new applications in medicine and food production. The SCOBY has been used as artificial skin to treat wounds or burnt skin [30]. Dehydrated SCOBY has potential in the production of kombucha berry gummies and SCOBY hamburgers due to its unique texture and flavour [12]. Therefore, it is important to determine and optimise the yield of the cellulosic pellicle from kombucha fermentation for these innovative applications.

The current challenges for industrial production of kombucha include the limited knowledge about the dynamics of its microbial community, such as the distribution of the microbial composition and lack of standardised fermentation process [6,31]. The diversity in the microbial composition of kombucha may be caused by differences in temperature, types of tea, climate, and also environmental conditions [7]. An improved understanding of the microbial composition of the SCOBY, and the changes in the levels of microorganisms during fermentation, will contribute to the better control of kombucha production.

This study determined the dominant microbial composition, and physico-chemical characteristics, of the Kombucha tea broth during fermentation and in the cellulosic pellicle. The microbial communities of kombucha and the cellulosic pellicle during fermentation were determined by culture-dependent methods and ribosomal genotyping. The present study provides an insight into the microbiological distribution of dominant AAB and yeast in the starter culture, cellulosic pellicle, and broth during the fermentation of traditional black tea kombucha.

## 2. Materials and Methods

### 2.1. Preparation of Fermented Kombucha

**Figure 1:**
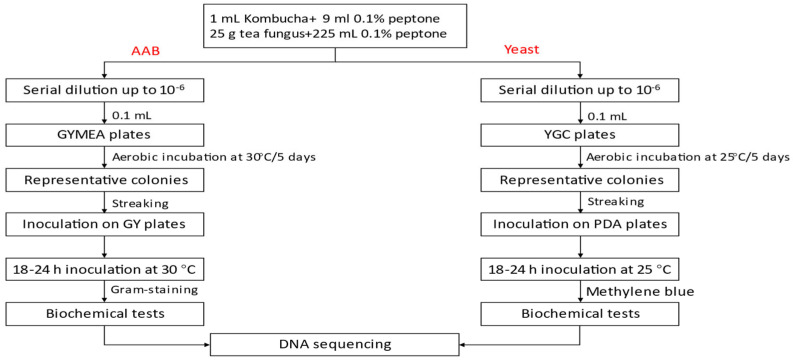
Various microbial analyses of kombucha tea broth and cellulosic pellicle. Adapted from Ref. [2].

The kombucha starter culture (cellulosic pellicle and liquid broth) was generated from kombucha commercially produced in New Zealand and available in local supermarkets in Auckland, New Zealand in 2021. The samples were transported to the Food Technology Department at Massey University, Auckland, under cold-chain maintained at 4 °C, after which they were stored at refrigeration temperature (4 °C) until required for further analysis (Figure 1).

### 2.2. Kombucha Fermentation Progress

Laboratory-scale fermented Kombucha was prepared using the modified methods of Greenwalt, Steinkraus, and Ledford [14] and Jayabalan, Malbasa, Loncar, Vitas, and Sathishkumar [15]. Sucrose (50 g/L) (white sugar, Chelsea, Auckland, New Zealand) was added into boiling water and mixed until completely dissolved. Black tea bags (Bell^®^, Auckland, New Zealand) (5 g/L) from a local supermarket were added to the mixture and allowed to infuse for 10 min then removed. The sugared tea was cooled to ambient temperature (~22 °C). The entire SCOBY (25 g/L) was gently placed on the surface of the sugared tea and a previous portion of fermented Kombucha tea broth (20%, *v*/*v*) was added to reduce the pH to prevent the growth of spoilage microorganisms, as well as promote the growth of fermenting microorganisms. The container was covered with a clean disposable cloth to keep out foreign matter. The fabric of the cloth still allowed adequate aeration of the broth which is essential for the growth of AAB. Fermentation was carried out at 22 °C for 14 days in a water bath (T100, Grant Optima, Cambridge, UK). Kombucha tea broth samples were collected for immediate physico-chemical and microbiological analyses at day 0, then every 2 days until the end of fermentation. The newly formed cellulosic pellicle (25 g), and samples (20 mL) of the fermented kombucha broth, were collected at the end of fermentation. The two components (broth and cellulose) were maintained in fresh sugared tea broth at 4 °C for the next fermentation.

### 2.3. Physico-Chemical Characteristics of Kombucha Broth Samples during Fermentation

#### 2.3.1. Determination of pH, Titratable Acidity (TA), and Total Soluble Solids (TSS) of Kombucha Tea Broth during Fermentation

The pH of the kombucha tea broth collected at different time points was measured using a Sartorius glass electrode pH meter (Model PB-11, Goettingen, Germany). Titratable acidity was determined by acid–base titration. Briefly, kombucha tea broth samples (20 mL) from different stages of fermentation were titrated against standardised 0.1 M sodium hydroxide to pH 8.2. The calculated TA was expressed as a percentage (%) of acetic acid per gramme of sample, as it is the major organic acid in kombucha (Equation (1)) [32]. The TSS of Kombucha tea broth samples were measured using a refractometer (Atago, pr-32 alpha, UK), according to Amarasinghe et al. [33].

(1) $$\%\;\mathrm{Titratable}\;\mathrm{acidity}\;\mathrm{of}\;\mathrm{acetic}\;\mathrm{acid}=\frac{\;V_{NaOH\;}\times M_{NaOH}\times 6.0053}{V_{sample}}$$

V_NaOH_ = volume of NaOH (mL)
M_NaOH_ = molarity of NaOH (M)
V_sample_ = volume of sample (mL)

#### 2.3.2. Determination of Wet Yield, Water Holding Capacity (WHC), Moisture Content, and Water Activity of Cellulosic Pellicle at the End of the Fermentation

The wet yield of the fresh pellicle produced during fermentation was determined using the method of Aung and Eun [34] with slight modifications. The weight of the initial mother pellicle used in the fermentation was measured at day 0 and at the end of fermentation (day 14). The wet yield of tea fungus was calculated using Equation (2).

(2) $$\mathrm{Wet}\;\mathrm{yield}\;(\%)=\frac{\mathrm{weight}\;\mathrm{of}\;\mathrm{total}\;\mathrm{fungus}\;\mathrm{at}\;\mathrm{day}\;14-\mathrm{weight}\;\mathrm{of}\;\mathrm{total}\;\mathrm{fungus}\;\mathrm{at}\;\mathrm{day}\;0\;}{\mathrm{weight}\;\mathrm{of}\;\mathrm{total}\;\mathrm{fungus}\;\mathrm{at}\;\mathrm{day}\;0}$$

The water holding capacity (WHC) and moisture content were determined using the methods of Avcioglu et al. [35] with slight modifications. The fresh cellulosic pellicle was cut into small pieces and mixed by blending (BB25EP, Waring, New York, NY, USA) for one minute. About 5–10 g of the pellicle were weighed into an aluminium pan and dried at 70 °C overnight. The WHC and moisture content were calculated using Equations (3) and (4), respectively.

(3) $$\mathrm{WHC}=\;\frac{\mathrm{weight}\;\mathrm{of}\;\mathrm{water}\;\mathrm{removed}\;\mathrm{during}\;\mathrm{drying}\;\left(g\right)}{\mathrm{dry}\;\mathrm{weight}\;\mathrm{of}\;\mathrm{tea}\;\mathrm{fungus}\;\left(g\right)}$$

(4) $$\mathrm{Moisture}\;\mathrm{content}=(\frac{w2-w3}{w2-w1})\times 100\%$$

W1: weight of empty container (g)
W2: weight of container and sample before drying (g)
W3: weight of container and sample after drying (g)

The water activity of the pellicle was measured using a water activity meter (AQUALAB 4TE, Pullman, WA, USA).

### 2.4. Isolation and Enumeration of AAB, LAB, and Yeast from Kombucha

Samples of both the cellulosic pellicle and kombucha tea, collected separately during fermentation, were used for the isolation and identification of AAB, LAB, and yeast. Serial dilutions (10–6) of the kombucha tea broth and pellicle were plated on appropriate microbiological media. Isolation of AAB from the Kombucha tea/pellicle was performed on modified glucose yeast extract mannitol ethanol acetic acid agar (GYMEA) containing 5% D-glucose (*w*/*v*) (ThermoFisher, Waltham, MA, USA), 2.5% mannitol (*w*/*v*) (ThermoFisher, Waltham, MA, USA), 1% yeast extract (*w*/*v*) (Sigma-Aldrich, St. Louis, MO, USA), and bacteriological agar (ThermoFisher, Waltham, MA, USA) using a slightly modified method from Wang, Rutherfurd-Markwick, Zhang, and Mutukumira [2]. Absolute ethanol (2% *v*/*v*) (Sigma–Aldrich, St. Louis, MO, USA) and glacial acetic acid (0.5% *v*/*v*) were added after autoclaving. The addition of cycloheximide (100 mg/L) and penicillin (1 mL/L) were intended to inhibit the growth of yeast and LAB, respectively. Samples for isolation of LAB and yeast were plated on De Man, Rogosa, and Sharpe (MRS) agar plates (ThermoFisher, Waltham, MA, USA) and yeast extract chloramphenicol (YGC) plates, respectively. The AAB were cultivated at 30 °C for 5–7 days; yeast was incubated at 25 °C for 5 days, aerobically. The potential LAB were incubated anaerobically at 37 °C for 72 h. Colonies of the grown microorganisms were enumerated, and results were expressed as colony-forming units (CFU)/mL or CFU/g. Representative AAB, LAB, and yeast isolates were purified by successive streaking on glucose yeast extract (GY), MRS and potato dextrose agar (PDA), respectively. The purified cultures were stored in 20% glycerol (*w*/*w*) at −80 °C for long term preservation, and on GY, MRS or PDA agar plates at 4 °C with monthly sub-culturing for routine use [36].

### 2.5. Phenotypic Characterisation of AAB and Yeast Isolated from Kombucha Tea Broth and Cellulosic Pellicle during Fermentation

#### 2.5.1. Phenotypic Characterisation of AAB

Three to five presumptive AAB isolates were randomly selected from the highest dilution (10^−6^) of kombucha tea broth at each time interval during fermentation [2]. A loopful of freshly purified AAB culture was used to determine phenotypic characterisation. The purified AAB isolates were plated on GY agar plates at 30 °C for 5–7 days and inoculated in the GY broth overnight with shaking at 170 rpm (Orbital Shakers, Ohaus, Parsippany, USA). The AAB culture was Gram-stained to examine cell morphology under oil immersion using the Carl Zeiss Transmission light microscope (Model HBO 50/AC, Oberkochen, Baden-Württeberg, Germany), and cell length of AAB isolates was measured using the Carl Zeiss AvixVision microscope software 4.8.1. Biochemical tests were then conducted on the Gram-negative isolates. Catalase positive tests were determined by formation of air bubbles on young, purified colonies after the addition of 1–2 drops of 6% H_2_O_2_ (*v*/*v*) (Sigma–Aldrich, St, Louis, MO, USA) [2,37]. Growth at different temperatures (25, 30, and 37 °C) was conducted on GY gar plates that were incubated aerobically for 5–7 days. Growth at low pH was determined in GY broth at pH 2 and pH 3 and incubated aerobically at 30 °C for 24 h. The oxidase test was conducted with oxidase strips (Oxoid, Cheshire, UK), and colour change was observed for 30 s [38]. The oxidation of ethanol to acetic acid, and then oxidation acetic acid to water and CO_2_, by AAB was carried out using Carr medium [39]. The ethanol tolerance test was performed on yeast extract agar plates containing 2%, 4%, 6%, 8%, and 10% (*v*/*v*) absolute ethanol [40]. Acid produced from sucrose, D-glucose, trehalose, and lactose was determined using the method of Arifuzzaman et al. [41] with slight modifications. Testing for the formation of cellulose by the AAB isolates was determined using the modified methods of previous reports [2,42,43]. The AAB isolates were inoculated in Hestrin–Schramm (HS) broth for 5–7 days at 30 °C. A small portion of the cellulose developed on the surface of the broth was carefully transferred into a 2 mL micro centrifuge tube mixed with 1 mL of 0.1 M NaOH solution and heated at 90 °C on a hotplate (Benchmark Scientific, Sayreville, NJ, USA) for 45 min. The oxidation of lactate and acetate of AAB isolates were determined using published methods [2,44]. Ketogenesis of glycerol to dihydroxyacetone (DHA) was performed with the modified method of Swings [45]. The AAB isolates were inoculated in glycerol yeast extract agar plates for 5–7 days at 30 °C; the surface of the agar plates was then flooded with Benedict’s reagent and incubated at room temperature for another 3 h to observe any colour change.

#### 2.5.2. Phenotypic Characteristics of Yeast

Three to five presumptive yeast isolates were randomly collected at each time interval during fermentation. The purified yeast isolates from kombucha tea broth and cellulosic pellicle were plated on PDA plates and incubated at 25 °C for 5 days. The cell morphology of yeast isolates was observed using the methylene blue staining method and the cell length was determined using the Carl Zeiss transmission light microscope (Model HBO 50/AC, Oberkochen, Baden-Württeberg, Germany) [2,46,47]. The young, fresh, and representative budding yeast isolates were then characterised using biochemical tests. The cycloheximide resistance test was conducted using the modified method of Kurtzman et al. [48]. The yeast isolates were streaked on the PDA plates containing 0.01% and 0.1% cycloheximide (*w*/*v*), respectively, and incubated at 25 °C for 5 days. The formation of colonies indicated resistance to the antibiotic. Growth at different temperatures was determined on PDA plates and yeast isolates were incubated at 25, 30, and 37 °C for 5 days, respectively. Resistance to high osmotic pressure was determined using the modified method of Kurtzman et al. [49]. Yeast isolates were inoculated on agar containing sodium chloride (10% *w*/*v*) and D-glucose (5% *w*/*v*) and incubated at 25 °C for 5 days. The growth of colonies suggested that the isolates had resistance to high osmotic pressure. The glacial acetic acid tolerance test was conducted on media containing (5% *w*/*v*) D-glucose, (1% *w*/*v*) tryptone, (1% *w*/*v*) yeast extract, (2% *w*/*v*) agar, and (1% *v*/*v*) glacial acetic acid. Growth at low pH (2 and 3) and acid produced from sucrose, D-glucose, trehalose, and lactose were previously described in Section 2.5.1.

### 2.6. Sequence Analysis of Ribosomal RNA Genes

Total DNA from representative AAB and yeast isolates were extracted using the Promega Wizard Genomic Purification Kit (ThermoFisher, Waltham, MA, USA) following the manufacturer’s instructions. The full-length 16S rRNA gene of AAB isolates was amplified by the polymerase chain reaction (PCR) using universal primer 27F and 1492R [2,50] and Taq DNA polymerase from Invitrogen (ThermoFisher, Auckland, New Zealand).

For yeast isolates, the internal transcribed spacer (ITS) regions were amplified by PCR using primers ITS1 and ITS4 [51]. The PCR products were purified with E.Z.N.A Cycle Pure Kit (Omega Bio-Tek InC., Norcross, GA, USA), and subsequent sequencing of the amplicons was performed by Massey Genome Service (Palmerston North, New Zealand), using primers 27F and 1492R, plus an internal primer 533F for 16S rRNA genes [52], and primers ITS1 and ITS4 for the ITS region. DNA sequences were processed using Geneious 9.0.5 (Biomatters Ltd., Auckland, New Zealand).

### 2.7. Statistical Analysis

The physicochemical and phenotypical characteristics experiments were performed in triplicate. Data on pH, titratable acidity, total soluble solids, yield, water activity, and moisture content were analysed by Microsoft Excel 2016 (Microsoft, Redmond, WA, USA) and represented as mean ± standard deviation. Data on pH, titratable acidity, total soluble solids, and viable cell counts were analysed by one-way analysis of variance (one-way ANOVA) using the IBM SPSS version 26 (IBM, New York, NY, USA) to determine significant differences between the means (*p* < 0.05). The DNA of the AAB and yeast was analysed by Geneious 9.1.8 (Biomatters, Ltd., Auckland, New Zealand). The closest homologues or best hits were identified by searching the EzBioCloud 16S rRNA gene database for AAB and ITS, and the database of NCBI using Basic Local Alignment Tool (BLAST) [53].

## 3. Results and Discussion

### 3.1. Physico-Chemical Characteristics of Kombucha Samples during Fermentation

#### Acidity and TSS of Kombucha during Fermentation

**Figure 2:**
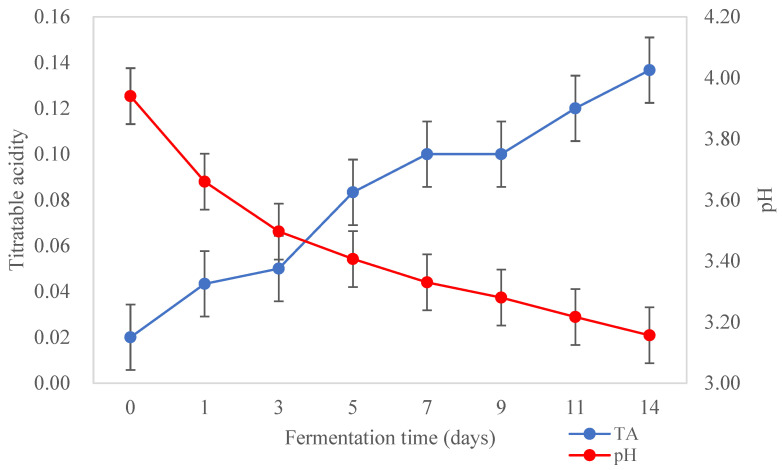
pH and titratable acidity of kombucha tea broth during fermentation for 14 days at 22 °C (*n* = 3).

The changes in acidity of kombucha tea broth during fermentation are shown in Figure 2. The pH of kombucha tea broth decreased from 3.94 ± 0.04 to 3.16 ± 0.01, and the TA increased from 0.02 ± 0.00 to 0.14 ± 0.13%, during the fermentation for 14 days at 22 °C (*p* < 0.05). A sharp decrease in pH was observed during the first three days of fermentation and then it decreased slowly to the lowest pH observed at the end of the fermentation (3.16 ± 0.01). Meanwhile, TA increased steadily throughout the fermentation as expected. During the fermentation, sucrose is hydrolysed by the yeast into glucose and fructose. The AAB then convert the two simple sugars into various organic acids such as acetic, gluconic, and glucuronic acids, which contribute to the acidity and functional activities such as the antimicrobial activity of kombucha [29]. Changes in the hydrogen ion concentration (pH) and TA during fermentation can affect the growth of fermenting microorganisms such as AAB and yeast in kombucha [3]. Low acid beverages (<pH 4) are considered to be microbiologically safe as they can prevent the growth of pathogenic microorganisms [14]. Currently, there is no standardised pH range for kombucha products. However, a very low pH may affect the overall sensory characteristics of kombucha, conferring a very sour flavour [54]. It is not recommended to produce kombucha with a pH < 2.5 as it may be harmful to some consumers due to the high acidity [55]. A pH range between 2.5 and 4.2 is considered desirable and safe for kombucha beverages [56].

**Figure 3:**
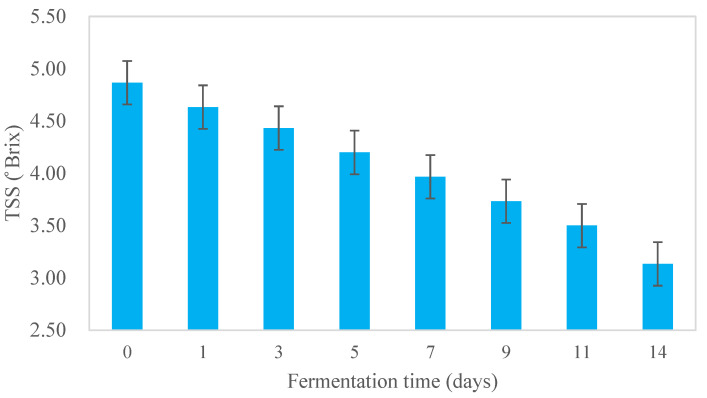
Total soluble solid (TSS) of kombucha tea broth during fermentation for 14 days at 22 °C (*n* = 3).

The TSS of Kombucha steadily decreased from 4.87 ± 0.06 to 3.13 ± 0.21° Brix throughout fermentation for 14 days (22 °C) (Figure 3) (*p* < 0.05). The reduction in TSS may be due to the microbial metabolism of sugar (sucrose) in the tea broth into a range of metabolites [15].

### 3.2. Wet Yield, WHC, Moisture Content, and Water Activity of Cellulosic Pellicle at the End of Fermentation

An approximately 2 cm thick transparent cellulosic layer was observed on the surface of the mother pellicle during kombucha fermentation. The AAB, which produce the cellulosic pellicle, are aerobic and therefore the pellicle floats on top of the broth [35]. The size of the cellulose pellicle gives an indication of the success of the fermentation process [5]. In this study, the wet yield of SCOBY was 27.75 ± 0.05 % by the end of the fermentation (Table 1). Bacterial cellulose has been widely used as an environmentally friendly nanomaterial in the cosmetic and food industries due to its high mechanical strength, high elasticity, high water-holding capacity, and chemical stability [35,57]. The WHC was 17.82 ± 0.16 g water/g dry pellicle, which was lower than cellulose produced by *Gluconacetobacter xylinus* (98.5 g water/g dry pellicle) in a previous study [58]. The WHC indicates the water retained by the cellulosic pellicle; a higher WHC results in a loose cellulosic structure which is desirable for nano-material applications [59].

### 3.3. Isolation and Enumeration of AAB and Yeast during Fermentation of Kombucha

**Figure 4:**
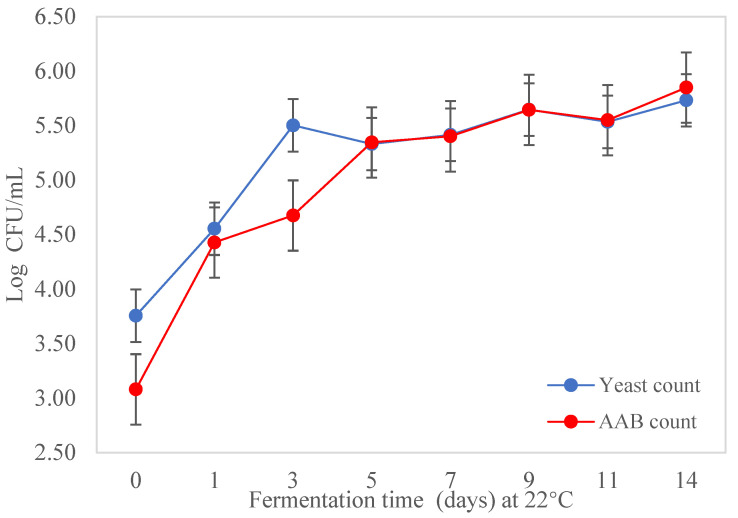
AAB and yeast count in kombucha tea broth during fermentation.

The changes (*p* < 0.05) in the microbial population during fermentation of the kombucha tea broth are shown in Figure 4. Yeast and AAB were found in the kombucha tea broth during fermentation and the pellicle. No LAB were present in the kombucha in this study. Kombucha was prepared using back-slopping fermentation, with the initial concentrations of yeast and AAB being 3.08 ± 0.42 log CFU/mL and 3.08 ± 0.48 log CFU/mL, respectively, in the kombucha tea broth. The microbial population of yeast and AAB in the kombucha tea broth increased by nearly 2 log CFU/mL during fermentation for 14 days (Figure 4). Although the initial cell counts of AAB were lower, the final population was slightly higher than the yeast count. Both AAB and yeast counts increased markedly from day 0 to day 5, and then slowly to the end of fermentation; yeast and AAB counts remained relatively similar from day 5 to day 11, with small fluctuations visible between day 9 and day 11. The cell counts from the cellulosic pellicle was 7.44 ± 0.31 log CFU/mL for AAB and 7.30 ± 0.06 log CFU/mL for yeast, which were higher than the number of microorganisms present in the tea broth (*p* < 0.05).

### 3.4. Phenotypic Characterisation of AAB and Yeast Isolated from the Kombucha Broth and Cellulosic Pellicle

#### 3.4.1. Morphology of AAB and Yeast Isolated from Kombucha Broth and Cellulosic Pellicle 

Sixty-eight strains (*n* = 29 AAB, *n* = 39 yeast) were isolated from the black tea kombucha during 14 days of fermentation. Based on the colony morphology, there was one group of AAB and two distinguishable groups of yeast isolated from the kombucha tea broth and pellicle. The AAB colonies had a circular shape, and smooth surfaces of brownish-red colour with an entire margin. The diameter of the AAB colonies ranged from 1.0 to 1.5 mm; the morphology of yeast grown on YGC plates is shown in Table 2. The different morphologies of the yeast colonies indicated that the fungal isolates may belong to different species.

Based on the Gram-staining reactions and cell morphology, 28 of the 29 AAB isolates from the kombucha tea broth and pellicle were Gram-negative [39]. All the presumptive AAB cells were rod-shaped with a similar cell length (~1.50 µm), and most occurred as singles. The cell morphology of AAB isolates in this study were similar to *Glucoacetobacter* or *Komagataeibacter* [60,61]. The formation of multipolar budding was observed from both groups (I and II) of yeast isolates. The average length of yeast cells ranged from 5.00 to 11.00 µm for the two groups of fungi, and were present singly, in pairs or in groups. The yeast cells were ovoidal to ellipsoidal shape in group I, and had a spherical shape in group II. The presumptive AAB isolates (*n* = 28) and yeast (*n* = 39) from the kombucha tea broth and pellicle were subjected to further characterisation.

#### 3.4.2. Phenotypic Characteristics of AAB and Yeast Isolated from Kombucha Tea Broth and Pellicle

The phenotypic and differential biochemical characteristics of the AAB isolates are shown in Table 3. Based on their biochemical profiles, the 28 AAB isolates were divided into six groups and, based on the data presented in Table 3, they were presumed to belong to the genus *Komagataeibacter.* All six groups of AAB isolates were oxidase-negative and catalase-positive, and were able to grow at 25, 30, and 37 °C after 5–7 days incubation. The bacteria were able to grow at pH 3 but not pH 2. All the AAB isolates produced cellulose after 5–7 days incubation at 30 °C without shaking. The transparent cellulose was not soluble after heating in aqueous NaOH. The production of cellulose is a typical characteristic of the species of *Gluconacectobacter* and *Komagataeibacter* [60]. All six groups of AAB isolates oxidised both lactate and acetate as the agar plates turned to blue.

All six groups of AAB isolates were able to grow on agar plates containing 2% (*v*/*v*) ethanol (alcoholic tolerance test). However, the six groups of AAB exhibited different growth profiles in media containing 4% to 10% (*v*/*v*) ethanol. Group IV isolates were able to grow in the presence of 10% ethanol, while group II and III were not able to grow in the presence of 4% (*v*/*v*) ethanol. The ethanol concentration of non-alcoholic kombucha from previous studies was reported to be between 0.2% and 1.1% (*v*/*v*) at the end of fermentation [3,6]. However, a higher alcohol content above the tolerance level of AAB has been reported post-fermentation of kombucha [2]. Therefore, it is important to monitor the fermentation process of kombucha as high levels of ethanol are undesirable for the growth of AAB.

All six groups of isolates oxidised ethanol to acetic acid as the agar turned to yellow after three days of incubation at 30 °C. The results suggest that groups II, IV, and VI oxidised acetic acid to water and CO_2_ after incubation for >5 days as the yellow became purple. The other three groups were not able to oxidise acetic acid probably because they were deficient in succinate dehydrogenase and α-ketoglutarate dehydrogenase [62]. All six groups were also able to oxidise glycerol to dihydroxyacetone (DHA). In the food industry, AAB convert glycerol to produce DHA, which imparts a crust-like taste in winemaking [63]. The presence of DHA in kombucha may confer a sweet, pleasant aroma, and enhances the production of cellulose that can positively impact the sensory characteristics of kombucha [2].

Table 4 shows the phenotypic characteristics of yeast isolated from the kombucha tea broth and cellulosic pellicle, with 4 isolates belonging to group II and 34 isolates to group I. The Group II yeast isolates were presumed to be the dominant yeast during the kombucha fermentation. The Group I isolates were not able to grow at 30 °C and 37 °C, while the group II isolates exhibited good growth at 30 °C and weak growth at 37 °C. Both the Group I and Group II isolates were able to grow on agar containing 0.01% cycloheximide. At higher cycloheximide concentrations, the Group I isolates showed weak growth. The Group I isolates had better growth on agar containing 1% glacial acetic acid than group II. The two groups of yeast isolates were able to produce acid from D-glucose and sucrose, but only Group II isolates produced acid from trehalose. These biochemical tests enable the differentiation of the yeast isolates into further groups [2]. However, it is difficult to identify yeast isolates to species or genus level based only on biochemical and physiological tests. Therefore, molecular methods were conducted for further identification.

### 3.5. Genetic Identification of Representative AAB and Yeast Isolates

Six representative AAB isolates from each group were subjected to sequence analysis of the full-length 16S rRNA gene. Similarly, three representative yeast isolates were subjected to ITS sequencing after biochemical testing. All six AAB isolates belonged to *Komagataeibacter rhaeticus,* showing 99.92% sequence similarity with the reference strain (Table 5). Only one nucleotide (nt) mismatch was found within the 1287 nt aligned sequence region. Therefore, in this study the dominant species present in both the tea broth and SCOBY was identified as *K. rhaeticus*.

All the 28 isolates obtained from different fermentation times in this study were isolated from New Zealand SCOBY in 2021, and there were minor differences in phenotypic characteristics compared with *K. rhzeticus* DST GL02^T^ [64]. This cellulose-producing strain was reported in Italian apple fruit, and its basonym was *Gluconacetobacter rhaeticus* [64]. It was then re-classified into the genus *Komagataibacter* in 2012 [65]. Strains AF-1, P1463, and K3 were isolated from kombucha on solid Hestrin–Schramm medium [43,57,66]. For example, Groups I, III, and IV were not able to oxidise ethanol to acetic acid, while reference strains can. Additionally, all six groups from our study exhibited different alcoholic tolerance in contrast to the reference strain [64,65]. Although these six group isolates belong to the same species, they are different strains and thus exhibited different biochemical profiles. The presence of *K. rhaeticus* contributes to the development of the cellulosic structure of kombucha as shown by the results from the cellulose formation test (Table 3).

The demand for bacterial cellulose (BC) has been rapidly increasing for applications in food, biomedicine, and cosmetics [43,57]. However, a low yield and high operational costs limit the production of BC [57]. Extensive research has been conducted to overcome these difficulties, including through isolating more productive strains, using lower cost carbon substrates, and optimising fermentation conditions [67]. Various strains from the genus *Komagataibacter* have been reported to be potential producers of BC including *K. xylinus, K. saccharivorans, K. hansenii*, and *K. rhaeticus* [67,68,69]. *K. rhaeticus* is a promising cellulose-producing resource, as it may have similar physicochemical characteristics to the BC produced by *K. xylinus* but with a higher yield under similar cultivation conditions [66]. Therefore, more research should be carried out to optimise the yield of BC synthesised by the *K. rhaeticus* isolated from this study and their impact on kombucha fermentation.

**Figure 5:**
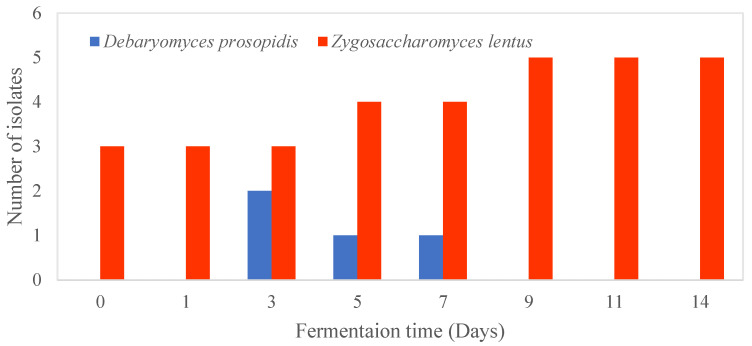
Distribution of yeast isolates in kombucha tea broth during fermentation for 14 days.

Based on the ITS sequence analysis, Group I yeast isolates belonged to *Zygosaccharomyces lentus* and showed a 99.84% sequence identity with the type strain CBS 8574. Only one mismatch was found with an ITS region of 634 nt in length. Group I isolates were obtained from both the cellulosic pellicle and kombucha tea broth at each sampling point during the fermentation (Figure 5). *Z. lentus* was firstly isolated from spoiled whole-orange drink and tentatively identified as *Zygosaccharomyces bailli* based on its carbohydrate assimilation profiles as analysed by API ID 32C system [70]. This species has been isolated from kombucha obtained in Germany and Ireland [71,72]. Strains of *Z. lentus* were distinguished from other strains of *Zygosaccharomyces* by their inability to grow above 25 °C; however, they could grow at low temperatures (4 °C) and low pH [73]. The inability of *Z. lentus* to grow under aerobic conditions was due to their sensitivity to oxygen [70]. Similarly, our yeast strains could grow at 25 °C but not 30 °C (Table 4). The higher cell counts of *Z. lentus* may be due to the favourable fermentation conditions used in this study (22 °C and low pH) under static conditions. As fermentation temperature can affect the balance of microorganisms in kombucha [74], a fermentation above 25 °C with the SCOBY used in this study is not recommended, as the dominant yeast species *Z. lentus* is not able to grow at that temperature. The strains isolated in this study were most similar in the phenotypic characteristics of reference strains IGC and 2406, especially in their ability to grow in medium containing 1% (*w*/*v*) acetic acid at low pH (pH 2 and 3) [73].

The Group II yeast isolates were present at the beginning (days 3, 5, and 7) of the fermentation in the kombucha tea broth only (Figure 5). Previous research also found that yeast was more diverse in the broth around day 7 of fermentation [75]. Although the culture-based methods used in this study may provide a good understanding of the physiological characteristics of the isolates, they are only effective in identifying the culturable microorganisms [76]. There may be unculturable yeast present in the SCOBY that cannot be detected using culture-based methods.

The Group II yeast belonged to *Debaryomyces prosopidis* with a 100% identity match to the type strain *D. prosopidis* JCM 9913. This species was considered a minor microbe from the kombucha yeast community in this study due its low proportion (10.3%). The phenotypic characteristics of the four strains isolated from this study agreed with the reference strains. The presence of *D. prosopidis* contributes positively to the texture and flavour characteristics of fermented meat products, possibly due to their high lipolytic activity and production of hexanal [77]. The presence of this species in kombucha may therefore contribute to its sensory profile. Recently, this species has been used to produce arabitol, a potential polyols sweetener from glycerol [78]. As glycerol is an intermediate metabolite during kombucha fermentation, the reaction between glycerol and *D. prosopidis* to produce arabitol may impact the sensory characteristics of kombucha to some extent. *Z. lentus* and *D. prosopidis* are rarely isolated from kombucha samples and therefore there is scant information about their role in the fermentation. Hence, more research should be conducted to investigate the interactions between the two yeast species found in this SCOBY and their impact on kombucha fermentation and metabolites.

## 4. Conclusions

All the AAB strains isolated from the black tea kombucha broth and pellicle during the fermentation were identified as *Komagataeibacter rhaeticus*, which are known for their cellulose production. The yeast species belonged to *Zygosacharomyces lentus* and *Debaryomyces prosopidis,* with the latter being dominant in the kombucha tea broth and pellicle. This study contributes important information on the microbiological composition of the cellulosic pellicle and physico-chemical characteristics of kombucha. Based on the findings of this study, it is recommended to ferment kombucha using the SCOBY from this study at <25 °C, as the growth of the dominant yeast species may be compromised. An improved knowledge of the microorganisms involved in kombucha fermentation can help manufacturers to better monitor the progress of fermentation, and aid in industrial scale-up to produce consistent high-quality products.

## Figures and Tables

**Table 1:** Wet yield, water holding capacity (WHC) moisture content, and water activity of cellulosic pellicle.

Characteristics	Cellulosic Pellicle
Wet yield (%)	27.75 ± 0.08
WHC (g water/g dry pellicle)	17.82 ± 0.16
Moisture content (%)	94.68 ± 0.05
Water activity at 21.45 °C	0.996 ± 0.002

**Table 2:** Appearance of yeast colonies grown on YGC agar plates.

Colony Group	Description of Appearance
Group I	Circular and slightly umbonate in the centre, white colour, smooth surface, and entire margin
Group II	Circular, brownish cream colour, flat and smooth surface

**Table 3:** Phenotypic characteristics of AAB isolated from kombucha broth and cellulosic pellicle.

Test	Group I	Group II	Group III	Group IV	Group V	Group VI
Oxidase	-	-	-	-	-	-
Catalase	+	+	+	+	+	+
Growth at different temperature						
Growth at 25 °C	+	+	+	+	+	+
Growth at 30 °C	+	+	+	+	+	+
Growth at 37 °C	+	+	+	+	+	+
Growth at different pH						
pH 2	-	-	-	-	-	-
pH 3	+	+	+	+	+	+
Cellulose formation	+	+	+	+	+	+
Growth without acetic acid	+	+	+	+	+	+
Oxidation of acetate	+	+	+	+	+	+
Oxidation of lactate	+	+	+	+	+	+
Alcoholic tolerance (*v*/*v*)						
2%	+	+	+	+	+	+
4%	+	-	-	+	+	+
6%	-	-	-	+	+	-
8%	-	-	-	+	-	-
10%	-	-	-	+	-	-
Acid produced from:						
D-glucose	+	+	+	+	+	+
Sucrose	-	-	-	-	-	-
Lactose	-	-	-	-	-	-
Trehalose	-	-	-	-	-	-
Oxidation of ethanol	-	+	-	+	-	-
Oxidation of ethanol to water and CO_2_	-	+	-	+	-	-
Ketogenesis of glycerol to DHA	+	+	+	+	+	+

Note: (+) indicated positive reaction and (-) indicated negative reaction. Experiments were replicated 3 times. Group 1 (*n* = 4), Group 2 (*n* = 8), Group 3 (*n* = 6), Group 4 (*n* = 2), Group 5 (*n* = 5), and Group 6 (*n* = 3).

**Table 4:** Phenotypic characteristics of yeast isolated from kombucha broth and cellulosic pellicles.

Tests	Group I	Group II
Growth in broth at 37 °C	-	w
Growth in broth at 30 °C	-	+
Growth in broth at 25 °C	+	+
Growth in medium containing 0.01% cycloheximide	+	+
Growth in medium containing 0.1% cycloheximide	w	+
Growth in medium containing 1% glacial acetic acid	+	w
Growth in medium containing 5% glucose and 10% NaCl	-	w
Growth in broth at pH 2	+	+
Growth in broth at pH 3	+	+
Acid produced from:		
D-glucose	+	+
Sucrose	+	+
Trehalose	-	+
Lactose	-	-

Note: (+) indicates positive reaction, (-) indicates negative reaction, and (w) indicates weak growth. Experiments were replicated 3 times. Group I (*n* = 35) and Group II (*n* = 4).

**Table 5:** Genetic identification of representative AAB and yeast isolates.

Representative Strains	Top Hit Taxon	Type Strain	GenBank Accession	% Similarity	Variation
AAB					
TFT1AAB26	*Komagataibacter rhaeticus*	DST GL02	AY180961	99.92	1/1292
Yeast					
GI: D3T3Y9	*Zygosaccharomyces lentus*	CBS 8574	NR_156001.1	99.84	1/634
GII: TFT1Y39	*Debaryomyces prosopidis*	JCM 9913	NR_077067.1	100.00	0/600

Note: the other five AAB strains genetically identified are D5T1AAB1, D7T2AAB10, D11T1AAB16, D11T2AAB17, and TFT3AAB29. GI: yeast Group I; GII: yeast Group II. An additional GII strain TFT3Y38 was also included in the analysis.

## Data Availability

Data are contained within the article.
